# Thermal conductivity of carbon nanotubes and graphene in epoxy nanofluids and nanocomposites

**DOI:** 10.1186/1556-276X-6-610

**Published:** 2011-12-01

**Authors:** Mario Martin-Gallego, Raquel Verdejo, Mohamed Khayet, Jose Maria Ortiz de Zarate, Mohamed Essalhi, Miguel Angel Lopez-Manchado

**Affiliations:** 1 Instituto de Ciencia y Tecnologia de Polimeros, ICTP-CSIC, Juan de la Cierva 3, Madrid, 28006, Spain; 2 Faculty of Physics, Complutense University, Madrid, 28040, Spain

**Keywords:** carbon nanotubes, graphene, nanocomposites, nanofluids, thermal conductivity

## Abstract

We employed an easy and direct method to measure the thermal conductivity of epoxy in the liquid (nanofluid) and solid (nanocomposite) states using both rodlike and platelet-like carbon-based nanostructures. Comparing the experimental results with the theoretical model, an anomalous enhancement was obtained with multiwall carbon nanotubes, probably due to their layered structure and lowest surface resistance. Puzzling results for functionalized graphene sheet nanocomposites suggest that phonon coupling of the vibrational modes of the graphene and of the polymeric matrix plays a dominant role on the thermal conductivities of the liquid and solid states.

**PACS**: 74.25.fc; 81.05.Qk; 81.07.Pr.

## Introduction

Due to the increasing importance of energy dissipation in the electronic industry, thermal conductivity of cured epoxy resins has been widely investigated over the years. One strategy to improve the thermal transport of epoxy resins has been the addition of highly conductive fillers, such as carbon-based or metallic fillers [1]. However, the effect of such additions on either the uncured system or the cure reaction of resins has not yet been fully established. The thermal conductivity of the uncured liquid resin plays an important role to define the variables involved in the transformation process, such as time, applied heat, or cooling time, which will then have a profound effect on the cross-link density and hence, on the final properties of the system. Thus, we aimed at studying the effect of two types of carbon-based nanofillers, in particular, nanotubes and graphene sheets, on the thermal conductivity of an uncured liquid epoxy resin.

There have been considerable interest and effort in the transport properties of carbon nanotube [CNT] filled polymer nanocomposites [2,3]. Electrical conductivity of epoxy nanocomposites increases by several orders of magnitude with CNT concentration [4]; this effect can be explained by the established percolation theory [5] with the shift from an insulator into a conductive material when a critical concentration of the conductive filler is reached, commonly known as percolation threshold. However, the thermal conductivity has shown at best linear enhancements with nanotube content with a lack of thermal percolation. The main reason for this fact is the relatively small thermal conductivity ratio (*K*_cnt_*/K*_matrix_) by comparison with the corresponding ratio of electrical conductivities [6].

Graphene is a two-dimensional carbon nanofiller with a one-atom-thick sheet of *sp*^2 ^bonded carbon atoms that are densely packed in a honeycomb crystal lattice [7,8]. Single layer graphene is predicted to have a remarkable performance, such as high thermal conductivity of 5,000 W/mK, which corresponds to the upper bound of the highest values reported for single-walled carbon nanotube bundles [9], high electrical conductivity of up to 6,000 S/cm [10], and superior mechanical properties with Young's modulus of 1 TPa and ultimate strength of 130 GPa [11]. In addition to these outstanding properties, the recent developments on graphene synthesis routes and on the understanding of their unique properties have prompted the development and study of graphene filled nanocomposites [12,13].

This communication analyzes the epoxy-nanofiller blend in the liquid state as a nanofluid and takes into consideration the current theories to explain its transport properties, particularly, the thermal conductivity. Some studies report large thermal enhancements by adding a small percentage of nanoparticles to a fluid [14]. This anomalous behavior, far away from the predicted data by standard theoretical models, is explained by several physical mechanisms like the Brownian motion of the particles or changes in the distribution of the molecules in the liquid state at the particle/liquid interface [15].

## Methods

### Materials

Diglycidyl ether of bisphenol-A epoxy resin (product number: 405493), diethylene triamine curing agent (D93856), and single-walled nanotubes [SWNTs] (519308; diameter 1.2 to 1.5 nm; length 2.5 μm; and specific surface area 1,300 m^2^/g) used in this study were purchased from Sigma-Aldrich (St. Louis, MO, USA), while multiwalled nanotubes [MWNTs] (diameter 40 nm; length 120 μm, and specific surface area 250 to 300 m^2^/g) were synthesized in-house by a chemical vapor deposition technique [16]. These MWNTs were then functionalized [f-MWNT] with a 3:1 concentrated H_2_SO_4_/HNO_3 _mixture refluxed at 120°C for 30 min and thoroughly washed with distilled water until neutral. Functionalized graphene sheets [FGS] were also synthesized in-house by the rapid thermal expansion of graphite oxide [GO] at 1,000°C under an inert atmosphere. This results in a high surface area carbon material consisting of graphene layers with residual hydroxyl, carbonyl, and epoxy groups. GO was synthesized from natural graphite flakes obtained from Sigma-Aldrich (St. Louis, MO, USA; universal grade, purum powder ≤ 0.1 mm, 200 mesh, 99.9995%), according to the Brödie method. Full characterization of the FGS used in this work is described elsewhere [17].

### Sample preparation and characterization

**Figure 1:**
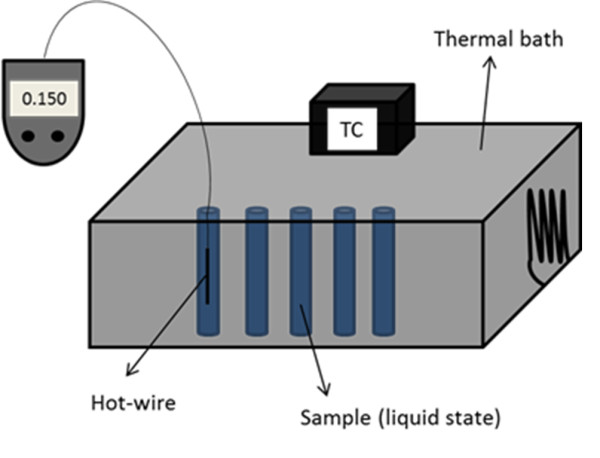
**Experimental setup**.

Nanoparticles were mixed under high shear in the resin for 8 h at room temperature to ensure a homogeneous dispersion. The thermal conductivity of the uncured nanofluids (the samples do not contain the curing agent) was measured with a KD2 probe (Decagon Devices Inc., Pullman, WA, USA), based on the hot wire technique, and consisting of a needle located inside the sample. As can be seen in the experimental setup (Figure 1), the hot wire enabled us to obtain the thermal conductivity in a direct and easy way. The needle had a waiting time of 30 s until the sample temperature was stable and heated up the sample for 30 s. Then, it was used to monitor the cooling rate and calculate the thermal conductivity with an accuracy of 5%. The measurements were carried out over a temperature range from 30°C to 60°C. In this range of temperature, no convection was present in the liquid. The results were the average of at least six measurements for each sample. On the other hand, the thermal conductivity of the cured samples was measured using a hot disk apparatus. This method was based on a heat balance in the steady state between the sample and the three disks of the apparatus that allowed us to calculate the thermal resistivity of the solid sample. Testing samples with different thicknesses were used to obtain the thermal conductivity of the material. The next protocol was followed to cure the formulations: the liquid formulations containing nanoparticles and epoxy resin were mixed with diethylene triamine in a stoichiometric ratio; the blends were degassed for 10 min in a vacuum chamber and casted in Teflon molds. Thermal treatments of 60 min at 70°C and 90 min at 130°C were applied to complete the curing reaction [18]. The morphology of the samples was observed using a Philips Tecnai 20 (Philips, Amsterdam, The Netherlands) transmission electron microscope at an acceleration voltage of 200 kV.

## Results and discussion

**Figure 2:**
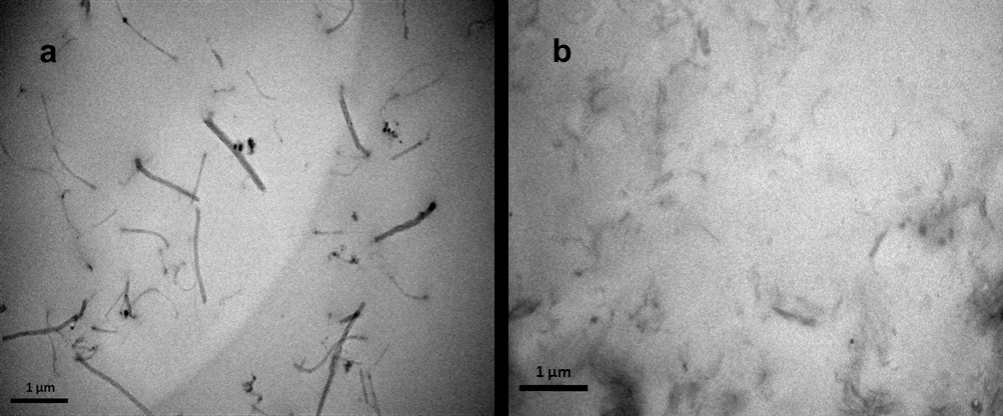
**TEM images of nanofluids**. (**a**) Resin loaded with 1 wt.% MWNTs and (**b**) with 1 wt.% FGS.

In Figure 2, we show the morphology of the uncured formulations with the highest concentrations of MWNT and FGS by transmission electron microscopy [TEM] analysis. In both cases, a homogeneous dispersion state was obtained.

The thermal conductivity [*K*] of CNTs depends on several factors such as the morphology, the chirality, the diameter and length of the tubes, the number of structural defects, and the specific surface area [19,20]. Thus, a description of the thermal conduction mechanisms is nontrivial. Liu et al. [21] reported a *K *for a SWNT and a MWNT of 2,400 W/mK and 1,400 W/mK, respectively, measured using the non contact Raman spectra shift method. The lower intrinsic conductivity of MWNTs was assigned to the fact that thermal transport mainly occurs by the outermost wall and by the existence of intertube Umklapp scattering processes. In addition, SWNTs exhibit a higher number of phonon vibrational modes and a lower defect density in relation to MWNTs, leading to a higher intrinsic *K *[22,23]. CNTs are characterized by a large aspect ratio and a huge surface area. It is assumed that the *K *of CNTs will be higher for CNTs with a greater aspect ratio [24]. In this study, both CNTs exhibit a similar aspect ratio, so this issue does not seem to affect the *K *of the nanofluid. Another factor that determines the *K *of CNTs is the presence of structural defects. Che et al. [25] revealed that the *K *of CNTs decreased with increasing defect concentration. Finally, the heat transfer mechanism of CNTs takes place with phonons and electrons and depends on their chirality [1]. However, to simplify the discussion, we assumed that the thermal conductance mainly occurs via a phonon conduction mechanism since the aim of this article is to provide a general description of the experimentally determined *K*.

Table 1 presents the *K *measurements of the nanofluids only at 30°C as no significant changes with the temperature were observed. The results indicate that MWNTs are the most effective carbon nanofillers to improve the *K *of liquid resins. Indeed, the *K *of the nanofluids gradually increased as a function of MWNT content, reaching a 70% improvement at 1 wt.% loading. The better performance of MWNTs can be due to their lower specific surface area [SSA], as compared with SWNTs, and to the presence of the internal layers which enable phonon conduction and hence minimize coupling losses. *K *of nanocomposites is sensitive to the quality of the interfacial bonding between the filler and the matrix, intimately related to a phonon coupling mechanism. This mechanism is influenced by numerous factors such as the length of free path for phonons, the boundary surface scattering, the number of vibration modes, and the resistance to heat flow at the interface, known as Kapitza resistance [26]. In general, the Kapitza resistance increases with the SSA, decreasing the efficiency of phonon transport.

**Table 1:** Thermal conductivity of epoxy nanofluids and nanocomposites

	*K*_liq_ (W/mK)	*K*_sol_ (W/mK)
Neat resin	0.150 ± 0.001	0.22 ± 0.07
0.2 wt.% MWNT	0.162 ± 0.004	-
0.4 wt.% MWNT	0.176 ± 0.009	-
0.6 wt.% MWNT	0.202 ± 0.004	0.29 ± 0.05
0.8 wt.% MWNT	0.220 ± 0.001	-
1 wt.% MWNT	0.250 ± 0.001	0.38 ± 0.07
0.6 wt.% f-MWNT	0.180 ± 0.001	-
0.6 wt.% SWNT	0.180 ± 0.001	-
1 wt.% FGS	0.150 ± 0.001	0.36 ± 0.04
1 wt.% Graphite	0.176 ± 0.005	-
1 wt.% GO	0.150 ± 0.001	-

A lower improvement was obtained with the acid-treated carbon nanotubes (f-MWNTs), even though the functionalization decreases the SSA of the nanotubes. This result can be explained by the presence of the functional groups, hydroxyl and carbonyl, that act as scattering points on the surface where phonons can be transferred from the nanotube crystalline structure into the insulating polymer matrix. This behavior has already been observed in cured resins [27,28], but not in the pre-cured state.

**Figure 3:**
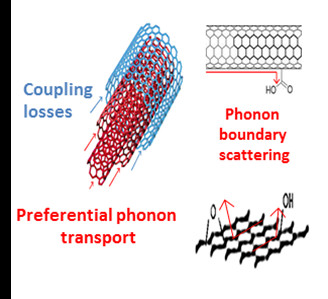
**Schema of the phonon coupling losses and the boundary phonon scattering at the nanoparticle interphase**.

The addition of nano-dispersed FGS caused no improvement of the *K *in the liquid resin. To better understand this result, we also measured dispersions of both the starting natural graphite and GO. The natural graphite showed a slight enhancement of *K*, while no improvement was observed in the oxidized system. These results support the previous discussion of the negative effect of the presence of functional groups on the nanoparticle surfaces [29], the presence of only one carbon layer, and the large SSA (see Figure 3). Hence, FGS cannot be considered as suitable fillers to enhance the *K *of liquid resins.

**Figure 4:**
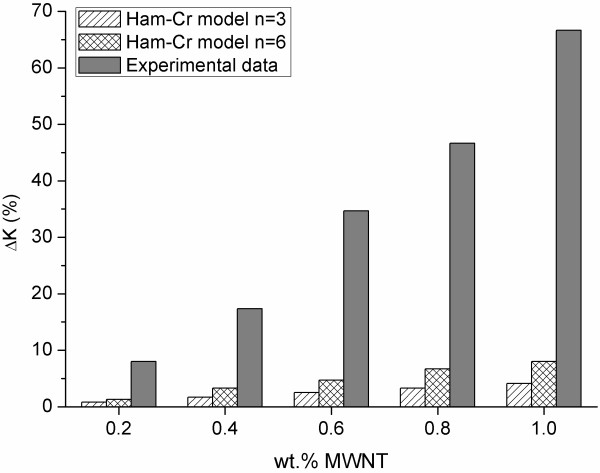
**Comparison of the measured data for MWNT nanofluids and the theoretical values**.

The enhancement in the *K *of MWNT nanofluids were fitted with the Hamilton-Crosser [30] model (Equation 1) traditionally used to predict the thermal enhancement of solid/liquid suspensions:

(1) $$\frac{K_{\text{e}}}{K_{\text{f}}}=1+\frac{n\left(\alpha -1\right)\phi }{\left(\alpha +n-1\right)-\left(\alpha -1\right)\phi }$$

where *K*_e _and *K*_f _are the effective thermal conductivities of the suspension and the base fluid, respectively; $\alpha =\frac{K_{\text{p}}}{K_{\text{f}}}$ is the *K *ratio, *K*_p _is the particle conductivity, *n *is the particle shape factor (*n *= 6 for cylinders, *n *= 3 for spheres in which case, Equation 1 reduces to the Maxwell model), and *φ *is the particle volume fraction calculated using the true density of the nanotubes [31]. The theoretical thermal conductivities are calculated with both values due to the bent conformation adopted by CNTs when dispersed in a matrix; thus, their shape factor would be between 3 and 6. Figure 4 compares the experimental results for the MWNT samples with the theoretical ones from the proposed model.

We observe an anomalous enhancement of the experimental *K*. This behavior could be related to two effects. The first effect is the presence of an organized structure of the molecules in the liquid state at the solid/liquid interface that facilities the coupling between the solid particles and the fluid [32]. The second effect could be contributions from the Brownian motions of the particles that modify the heat transfer in the fluid [33].

**Figure 5:**
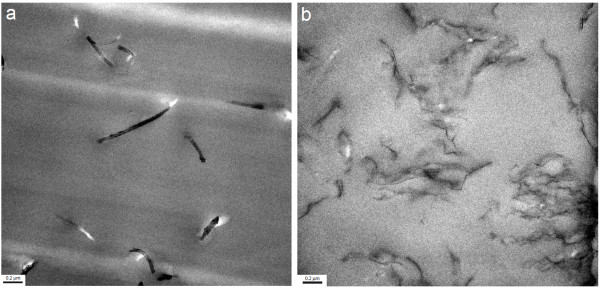
**TEM images of cured epoxy samples**. (**a**) Resin loaded with 1 wt.% MWNT and (**b**) with 1 wt.% FGS.

We finally measured the *K *of the cured samples for some of the nanocomposites with a classical hot-plate apparatus. TEM microphotographs show a finely and homogeneous dispersion of the carbon nanostructures, MWNTs, and FGS in the cured epoxy samples (Figure 5). The improvements obtained for the cured MWNT nanocomposites are approximately the same as those in the liquid state and are in agreement with the data found in the literature [27,28,34]. The cured FGS sample revealed a similar enhancement of the *K *as the MWNT sample. This increase of *K *in cured epoxy resin due to the addition of FGS has already been reported [35,36], but not the uncured/cured transition. This transition suggests that the results can be attributed to the differences in the media surrounding the nanoparticles when the resin is in the liquid or solid state. While the FGS are dispersed in a liquid media, they are not able to transfer the heat because the vibrational modes are not compatible. However, when the FGS are surrounded by the more rigid cured matrix, the differences between the frequencies of vibrational modes are smaller and enable phonon coupling. This result corroborates a current theory postulating that the dominant factor in nanocomposite heat conduction is the low frequency modes and their coupling with high vibrational modes at the interface [37,38]. This transition does not exist in the MWNT samples because the phonon transport through the inner tubes should be relatively unperturbed by the surrounding matrix.

## Conclusions

We employed an easy and direct method based on the hot wire technique to measure the thermal conductivity of epoxy nanofluids. We also studied the differences in heat conduction mechanisms using graphene sheets and different types of CNTs analyzing the role of surface functionalization and resistance to heat flow at the interface in the thermal conductivity. The results show that the layered structure of MWNTs enables an efficient phonon transport through the inner layers, while SWNTs present a higher resistance to heat flow at the interface due to its higher SSA, and f-MWNTs have functional groups on their surface acting as scattering points for the phonon transport. The dominant role of coupling vibrational modes between the matrix and the filler is evident in the case of FGS which induces a transition from a non thermal conductive nanofluid into a thermal-conductive nanocomposite in the solid state.

## Competing interests

The authors declare that they have no competing interests.

## Authors' contributions

The work presented here was carried out in collaboration between all authors. MMG carried out the synthesis and characterization of nanofillers and nanocomposites, participated in the discussion, and drafted the manuscript. RV helped in nanocomposite preparation, participated in the discussion, and revised the manuscript. MK supervised the thermal conductivity measurements and revised the manuscript providing important intellectual contents. ME performed the experimental setup. JMOZ provided the software to acquire the experimental data and contributed to the discussions of the results. MALM designed and coordinated the study, led the discussion of the results, and revised the manuscript. All authors read and approved the final manuscript.
